# Supplementary figures and images for: VAPA mediates lipid exchange between Leishmania amazonensis and host macrophages
Source: PLoS Pathog. 2025 Mar 31;21(3):e1012636. doi: 10.1371/journal.ppat.1012636 (PMC11981147; doi:10.1371/journal.ppat.1012636)

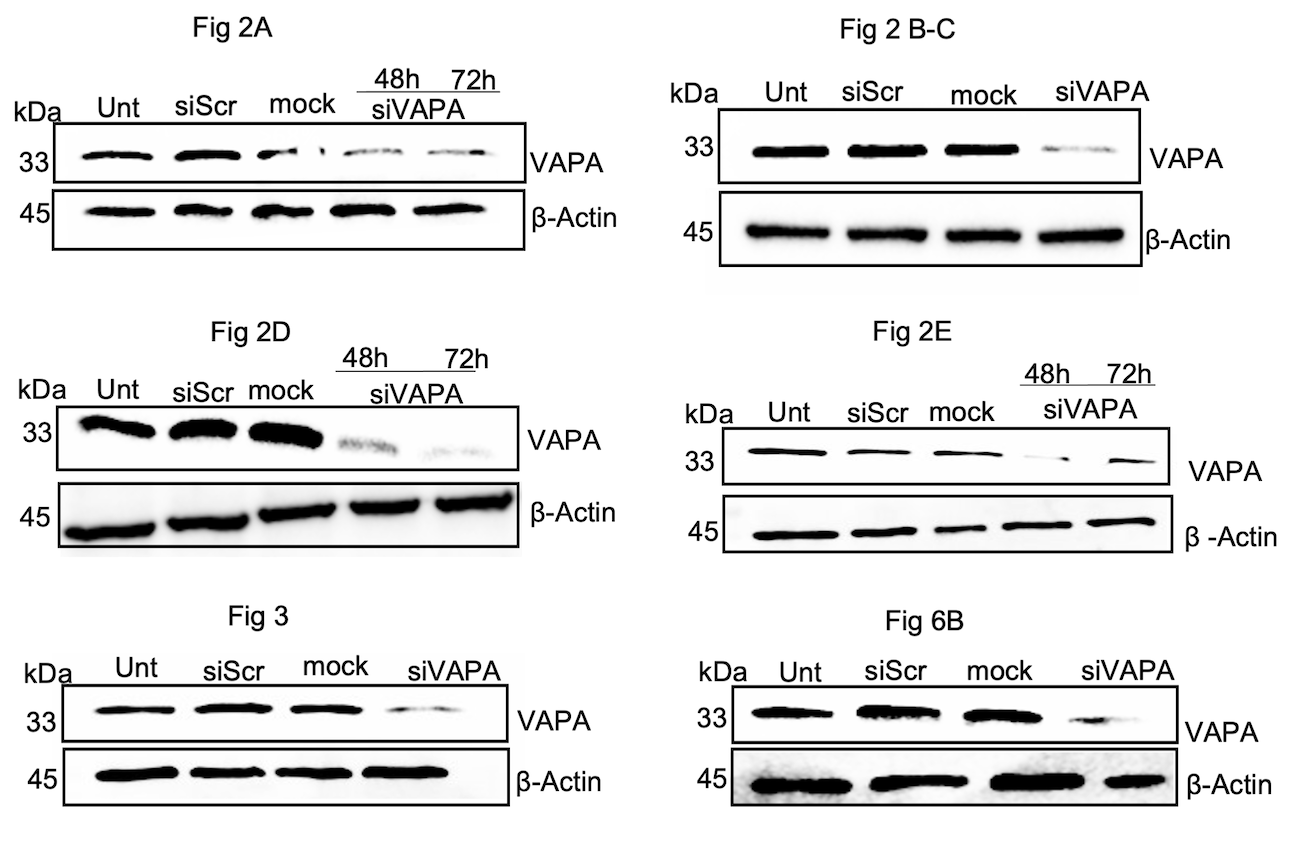

Supplement: S1 Fig — Efficacy of siRNA-mediated knockdown of VAPA in BMM. Representative Western blots of VAPA levels in untreated BMM (unt), BMM treated with scrambled siRNA (siScr), mock transfected BMM (mock) and BMM treated with siRNA to VAPA (siVAPA). Levels of β-actin were used as controls. Blots for the levels of VAPA for the results shown in Figs 2A-E, 3A, 3B and 6B. (TIFF) [file ppat.1012636.s001.tiff]

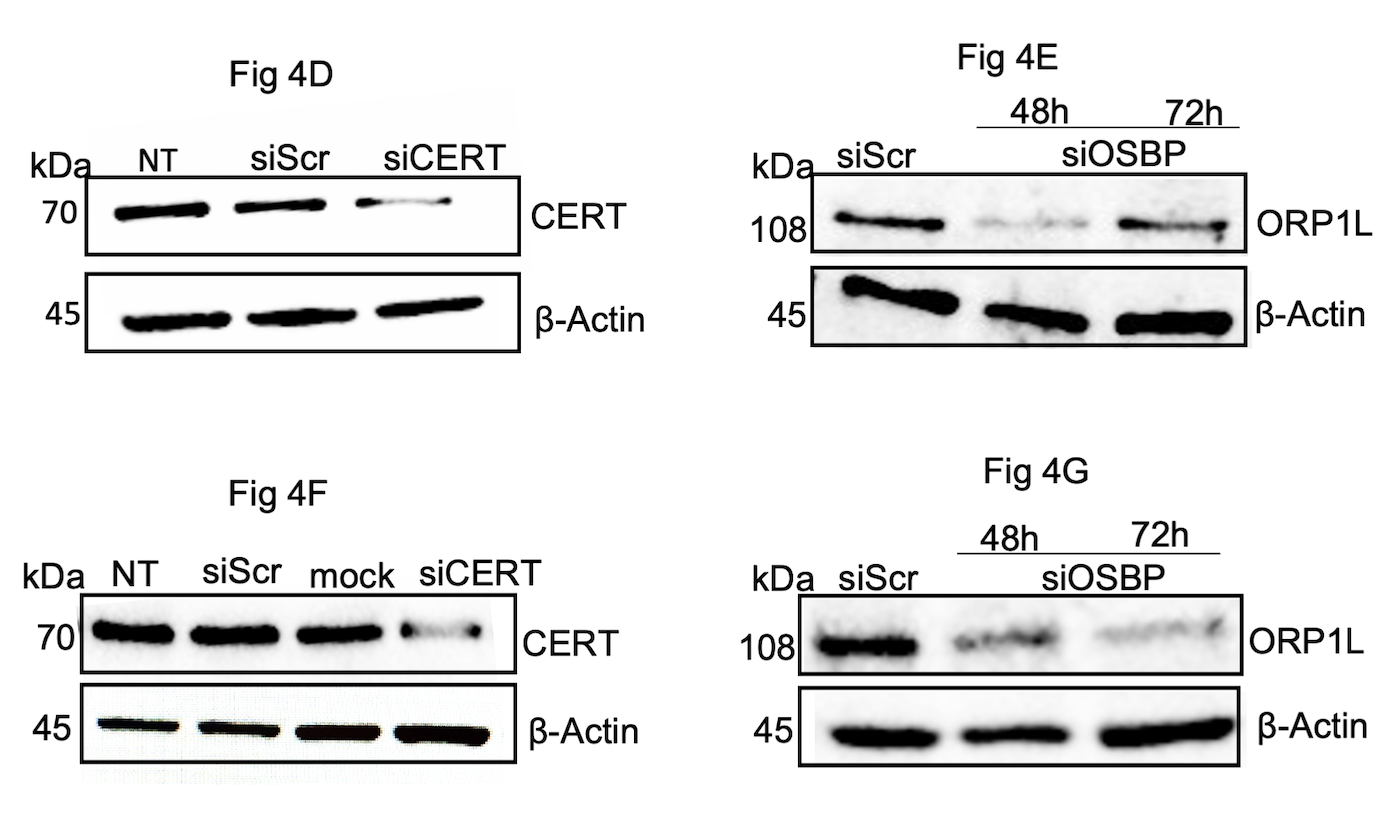

Supplement: S2 Fig — Efficacy of siRNA-mediated knockdown of CERT and ORP1L in BMM. Representative Western blots of CERT and ORP1L levels in untreated BMM (unt), BMM treated with scrambled siRNA (siScr), mock transfected BMM (mock) and BMM treated with either siRNA to CERT (siCERT) or siRNA to ORP1L (siOSBP). Levels of β-actin were used as controls. Blots for the levels of CERT for the results shown in Fig 4D and 4F and in for the levels of ORP1L for the results shown in Fig 4E and 4G. (TIFF) [file ppat.1012636.s002.tiff]

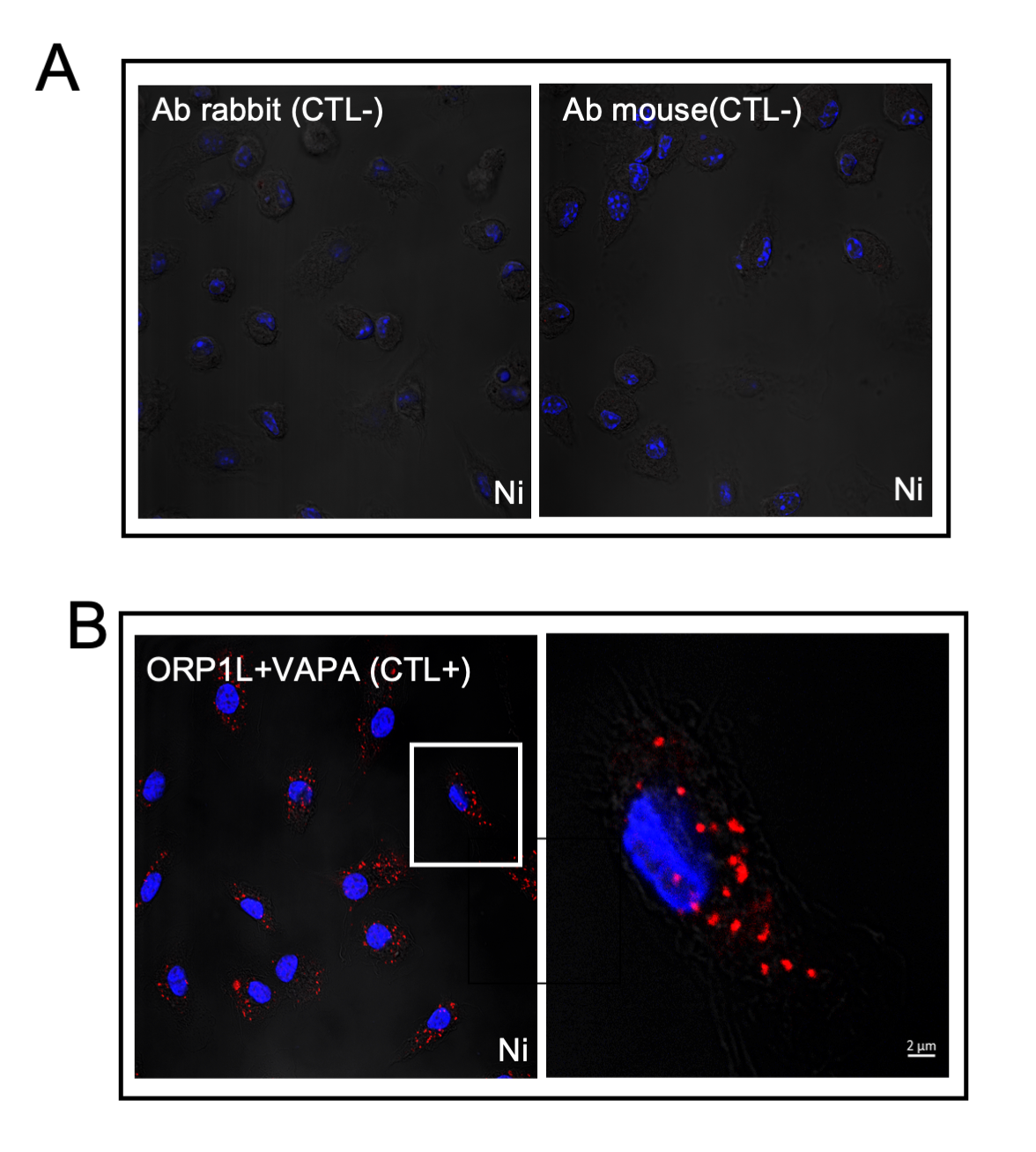

Supplement: S3 Fig — Controls for the proximity ligation assays. (A) To determine the background signal of proximity ligation assays, adherent BMM were incubated with only one of the individual primary antibodies at a time. Proximity ligation assays were performed on uninfected BMM in the absence of the secondary antibodies against either the anti-rabbit primary antibodies or the anti-mouse primary antibodies. (B). Positive control for the proximity ligation assays was performed on uninfected BMM using primary antibodies against ORP1L and VAPA. Red dots represent VAPA-ORP1L in situ complexes. DNA is in blue. (TIFF) [file ppat.1012636.s003.tiff]

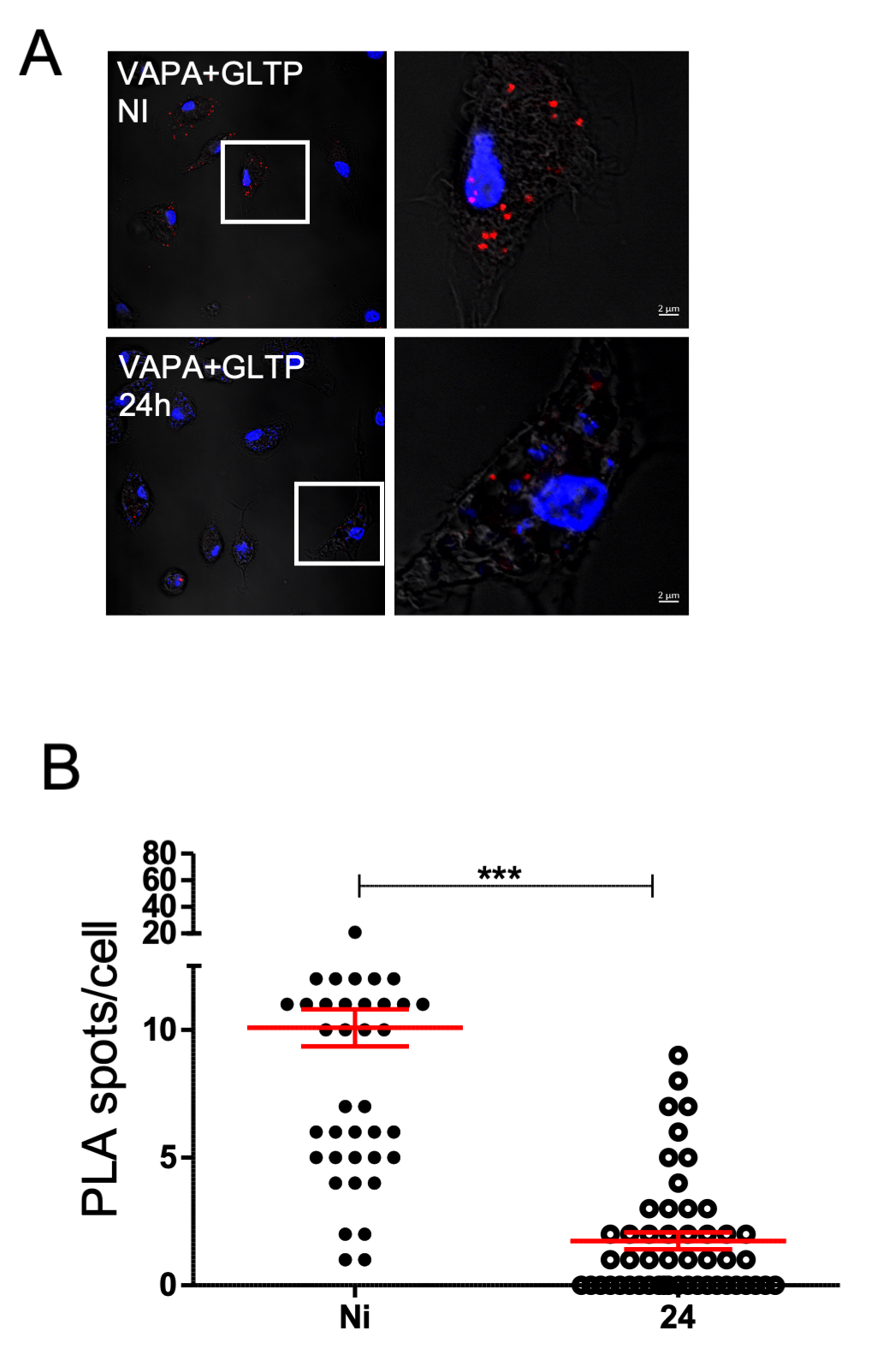

Supplement: S4 Fig — Disruption of VAPA-GLTP in situ complexes by L. amazonensis. (A) BMMs were infected or not with either L. amazonensis metacyclic promastigotes. At 24 h post-infection, VAPA-GLTP in situ complexes were detected by proximity ligation and visualized by confocal immunofluorescence microscopy (red dots). DNA is in blue. (B) Quantification of in situ complexes for VAPA-GLTP in uninfected BMM and in BMM infected with L. amazonensis. Data are presented as clouds with means ± standard deviations (SD) of values from three independent experiments for a total of 75 cells in each group. ***, P ≤ 001. (TIFF) [file ppat.1012636.s004.tiff]
